# Resting-state functional magnetic resonance imaging indices are related to electrophysiological dysfunction in degenerative cervical myelopathy

**DOI:** 10.1038/s41598-024-53051-x

**Published:** 2024-01-29

**Authors:** Hironobu Akimoto, Hidenori Suzuki, Shigeyuki Kan, Masahiro Funaba, Norihiro Nishida, Kazuhiro Fujimoto, Hiroaki Ikeda, Teppei Yonezawa, Kojiro Ikushima, Yoichiro Shimizu, Toshio Matsubara, Kenichiro Harada, Shin Nakagawa, Takashi Sakai

**Affiliations:** 1 Department of Orthopedic Surgery, Yamaguchi University Graduate School of Medicine, 1-1-1 Minami-Kogushi, Ube, Yamaguchi 755-8505 Japan; 2 Department of Psychiatry and Neurosciences, Graduate School of Biomedical and Health Science, Hiroshima University, Hiroshima, Hiroshima 734-8553 Japan; 3 Department of Anesthesiology and Intensive Care Medicine, Osaka University Graduate School of Medicine, Suita, Osaka 565-0871 Japan; 4 Department of Radiological Technology, Yamaguchi University Hospital, 1-1-1 Minami-Kogushi, Ube, Yamaguchi 755-8505 Japan; 5 Division of Neuropsychiatry, Department of Neuroscience, Yamaguchi University Graduate School of Medicine, Ube, Yamaguchi 755-8505 Japan

**Keywords:** Sensorimotor processing, Somatosensory system

## Abstract

The age-related degenerative pathologies of the cervical spinal column that comprise degenerative cervical myelopathy (DCM) cause myelopathy due spinal cord compression. Functional neurological assessment of DCM can potentially reveal the severity and pathological mechanism of DCM. However, functional assessment by conventional MRI remains difficult. This study used resting-state functional MRI (rs-fMRI) to investigate the relationship between functional connectivity (FC) strength and neurophysiological indices and examined the feasibility of functional assessment by FC for DCM. Preoperatively, 34 patients with DCM underwent rs-fMRI scans. Preoperative central motor conduction time (CMCT) reflecting motor functional disability and intraoperative somatosensory evoked potentials (SEP) reflecting sensory functional disability were recorded as electrophysiological indices of severity of the cervical spinal cord impairment. We performed seed-to-voxel FC analysis and correlation analyses between FC strength and the two electrophysiological indices. We found that FC strength between the primary motor cortex and the precuneus correlated significantly positively with CMCT, and that between the lateral part of the sensorimotor cortex and the lateral occipital cortex also showed a significantly positive correlation with SEP amplitudes. These results suggest that we can evaluate neurological and electrophysiological severity in patients with DCM by analyzing FC strengths between certain brain regions.

## Introduction

Degenerative cervical myelopathy includes several age-related degenerative pathologies affecting the cervical spinal column. Compression of the spinal cord causes myelopathies that lead to motor and sensory dysfunction in the upper and lower limbs, such as osteoarthritic degeneration (e.g., cervical spondylosis) and ligamentous abnormalities (posterior longitudinal ligament or ligamentum flavum ossification)^1–4^. The spectrum of cervical disorders caused by DCM typically cause progressive and often irreversible deterioration and loss of function in the cervical spinal cord^1–4^. Patients clinically assessed to have DCM report various symptoms such as neck pain, range of motion impairment of the limbs, clumsy hands and numbness, loss of manual dexterity, paresthesia, gait disorder, and bladder and bowel disturbances^5–7^, indicating multiple etiologies of DCM pathology. Many kinds of neurological, physical, and electrophysiological examinations have been conducted to evaluate the severity of neurological deficits and surgical indications^8–13^. Therefore, to diagnose DCM precisely, we need to comprehensively understand the clinical history and multiple test results, such as physical and electrophysiological testing and imaging findings from kinematics X-ray, magnetic resonance imaging (MRI), and computed tomography^14,15^. However, morphological imaging techniques are currently not considered suitable methods for DCM diagnosis because abnormalities reflected on these modalities do not reflect neurological function and neurological deficits in patients with DCM^16–18^. In addition to those conventional methods, diffusion tensor imaging (DTI), which can evaluate the condition of the white matter in the central nervous system, has been recently regarded as a promising assessment tool for the severity of fiber damage and neurological function in DCM^19–21^. The other article reported that artificial intelligence (AI) methods are feasible and effective for DTI analysis for the prognosis of DCM^22^. However, tractography, including DTI, is not considered a standard approach due to its limitations, variability of obtained data, and lack of standardization of image acquisition parameters, as present^20,21,23^. Analysis of DTI features is also complicated and time consuming. Moreover, it has pitfalls that limit the anatomical accuracy of this technique^23^.

Resting-state functional MRI (rs-fMRI) is a new neuroimaging paradigm that has attracted attention in basic and clinical medicine to resolve these issues mentioned above. Rs-fMRI can measure spontaneous regional brain activity based on the blood oxygen level-dependent effect, and this paradigm may potentially provide a biomarker reflecting functional disability or specific neurological, sensory, and motor dysfunction^24–28^. Recently, multiple independent rs-fMRI studies reported alterations in spontaneous brain activity in patients with DCM^29–42^. Furthermore, several studies attempted to predict postoperative recovery by using preoperative indices of brain activity in patients with DCM. These studies focused on the sensorimotor areas, subcortical regions, limbic system, and the visual cortex^33,34,37,40^ and showed that spontaneous brain activity in these brain regions was related to the pathology of DCM^30–32^, thus suggesting that rs-fMRI can potentially reveal the pathophysiological mechanism of DCM.

Electrophysiological examinations are the most objective and quantitative assessments available to examine neurological function in patients with DCM^43–49^. Central motor conduction time (CMCT) and sensory evoked potentials (SEP) have been established as indices of motor and sensory function, respectively, in the clinical diagnosis of DCM. In addition, these indices are useful to examine the severity of DCM and any neurological deficit^43–49^. Abnormal CMCT and SEP are reportedly related to the presence of spinal cord compression in patients with DCM^12,43–50^. Previous CMCT studies showed this index to be an objective and sensitive measure for the assessment of central motor pathway condition^12,43–50^. Disruption of fast monosynaptic neurons in the lateral corticospinal tract during the early stage of cord compression can prolong CMCT. Therefore, CMCT can be used to determine the presence of myelopathy with high sensitivity^43^. SEP can detect sensory tract damage with high sensitivity^51,52^. Thus, it is widely used as an intraoperative neuromonitoring tool for spinal surgery to check whether surgical procedures have injured the sensory tract^53^. However, these electrophysiological tests take about an hour for one session and are invasive. Furthermore, facilities capable of conducting such specialized tests are extremely limited, often available only in specific university hospitals^13,43–47^. In contrast, rs-fMRI is quickly and easily performed without discomfort for DCM patients. Moreover, it can be conducted anywhere with access to an MRI scanner. Therefore, rs-fMRI appears a promising alternative for these electrophysiological tests.

In this study, we used these electrophysiological indices as objective and quantitative measures of neurological dysfunction and neurophysiological status in patients with DCM, and we comprehensively investigated the relationship between CMCT/SEP and the following measures of spontaneous brain activity, i.e., the fractional amplitude of low-frequency fluctuations (fALFF) and functional connectivity (FC), in patients with DCM. Correlation analysis revealed a significant correlation of FC with CMCT and SEP, suggesting that rs-fMRI measures could provide a quantitative biomarker reflecting the severity of motor and sensory dysfunction in patients with DCM.

## Results

### The demographic and clinical information

Thirty-four patients with DCM and 21 HCs participated in this study (Table 3). There was no significant age difference between patients with DCM and HCs (p = 0.995). In addition, there was no significant sex difference between patients with DCM and HCs (p = 0.94). The mean total JOA score in DCM patients was 11.0 ± 2.4. The medications taken by each patient ware also shown in Table 3.

We did not perform the CMCT measurement in patients who had a cardiac pacemaker or intracardiac lines or metal in body because of the contraindication^13,43–47^. As a result, we measured CMCT from 33 patients with DCM (Table 3). We could not examine SEP amplitudes for 14 patients because SEP could not be measured in patients with very severe paralysis. Therefore, we measured SEP amplitude to 20 patients with DCM (Table 3).

### Data quality

Rs-fMRI scans were performed on all patients with DCM. The number of excluded fMRI volumes in the scrubbing procedure between the patients with DCM and the HC was not significantly different (DCM, 4.52 ± 6.51; HC, 3.71 ± 5.25; p = 0.613). Body movements in each direction (x, y, z, pitch, roll, yaw) were also not significantly different between the patients with DCM and HC during rs-fMRI scanning (DCM: x = 0.0329 ± 0.0378, y = 0.0886 ± 0.0459, z = 0.0913 ± 0.0636, pitch = 0.00126 ± 0.0007, roll = 0.000625 ± 0.000707, yaw = 0.0005 ± 0.000466; HC: x = 0.0189 ± 0.0096, y = 0.0666 ± 0.0338, z = 0.0634 ± 0.0368, pitch = 0.00109 ± 0.00082, roll = 0.000534 ± 0.000202, yaw = 0.000354 ± 0.000142; all p-values > 0.0627).

### Comparison of the fALFF between patients with DCM and the healthy control group

We compared fALFF in the < 0.1 Hz low-frequency band to that in a full-frequency band of fMRI signals between patients with DCM and healthy controls (HC). This measure reflects the magnitude of spontaneous regional brain activity^12,50^. The patients with DCM showed significant increase in fALFF in the occipital pole and in the lateral occipital cortex superior division compared with those in the HC (Table 1). Furthermore, patients with DCM showed a significant decrease in fALFF in the cerebellar hemisphere, left superior parietal lobule, right frontal pole, right central opercular cortex, left thalamus, and the right inferior temporal gyrus posterior division (Table 1). However, we found no significant correlations between fALFF values and CMCT (all clusters were smaller than 38 voxels corresponding to that all p-values > 0.684) or SEP amplitude (all clusters were smaller than 70 voxels corresponding to that all p-values > 0.090). We showed the result of the correlation analysis between fALFF values and CMCT or SEP amplitude in Supplemental Table S1.

**Table 1:** Differences in fALFF between patients with degenerative cervical myelopathy and healthy control.

Comparison		Regions	Cluster size (voxel)	P value	T value	x	y	z
DCM > HC	Cluster 1	Occipital pole R, Lateral occipital cotex superior division R	1174	0.0000	7.29	22	− 94	− 12
	Cluster 2	Lateral occipital cortex superior division L, Occipital pole L	881	0.0000	6.07	− 10	− 88	38
	Cluster 3	Occipital pole L, Lateral occipital cortex inferior division L	816	0.0000	6.34	− 30	− 96	− 14
	Cluster 4	Lateral occipital cortex superior division R, Occipital pole R	59	0.0420	4.13	14	− 80	48
HC > DCM	Cluster 1	Cerebelum 9 R, Cerebelum 8 R	213	0.0001	5.28	10	− 54	− 44
	Cluster 2	Cerebelum 9 L, Cerebelum 8 L	161	0.0009	4.48	− 8	− 56	− 42
	Cluster 3	Superior parietal lobule L, Precuneus cortex	121	0.0042	4.35	− 16	− 54	52
	Cluster 4	Crus posterius capsulae internae R	110	0.0060	5.25	28	− 36	36
	Cluster 5	Frontal pole R, Frontal orbital cotex R	104	0.0069	4.64	26	34	− 18
	Cluster 6	Central opercular cortex R	73	0.0287	5.03	32	2	20
	Cluster 7	Thalamus L	66	0.0376	4.87	− 12	− 26	6
	Cluster 8	Crus posterius capsulae internae R	60	0.0420	4.72	36	− 48	20
	Cluster 9	Inferior temporal gyrus posterior division R (temporoccipital part R)	59	0.0420	4.90	50	− 38	− 18

x, y, and z represent the local maximum in a cluster in the MNI coordinate. T value is the t value of a local maximum.

Cluster-level threshold was p < 0.05 (false discovery rate corrected).

*fALFF* fractional amplitude of low-frequency fluctuation, *DCM* degenerative cervical myelopathy, *HC* healthy control, *R* right, *L* left.

### Correlation between FC strength and CMCT in patients with DCM

**Figure 1:**
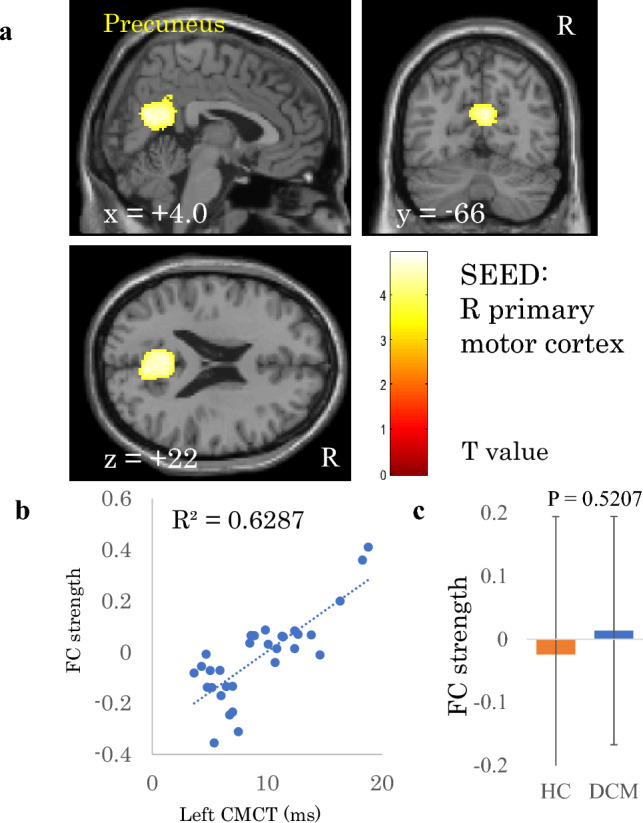
Correlation between left central motor conduction time (CMCT) and functional connectivity (FC) strength of the right primary motor cortex. (**a**) A cluster showing a significant positive correlation between left CMCT and FC strength of the right primary motor cortex, **b**) scatter plot of left CMCT versus FC strength, and **c**) comparison of FC strengths between patients with degenerative cervical myelopathy (DCM) and healthy controls (HC). The dashed line in panel **b** is the regression line. Error bars in panel **c** represent standard deviations.

In addition to fALFF, we investigated correlations between FC strength and the electrophysiological indices. We found that FC strength between the right primary motor cortex and the right precuneus correlated significantly and positively with CMCT (r = 0.793, Fig. 1, Table 2). However, the strength of this FC was not significantly different between the patients with DCM and the HC (p = 0.5207).

**Table 2:** Correlation between FC strength and electrophysiological index in patients with DCM.

Electrophysiological index	Seeds/Sources	Region	Cluster size	P value	T value	x	y	z
R CMCT	L primary motor cortex (BA 4)			n.s				
L CMCT	R primary motor cortex (BA 4)	Precneous	830	0.0016	4.94	4	− 66	22
R SEP amplitude	L primary somatosensory cortex (BA 1, 2, 3)			n.s				
	L thalamaus	L precentral gyrus	249	0.0037	5.74	− 34	0	40
	L lateral part of sensorimotor network			n.s				
L SEP amplitude	R primary somatosensory cortex (BA 1, 2, 3)			n.s				
	R thalamaus			n.s				
	R lateral part of sensorimotor network	R lateral occipital cortex	301	0.0006	− 5.2	44	− 70	− 16
		Cingulate gyrus anterior division	149	0.0056	5.34	4	4	42

x, y, and z represent the local maximum in a cluster in the MNI coordinate. T value is the t value of a local maximum.

Cluster-level threshold was p < 0.05 (false discovery rate corrected). Bonferroni correction was further applied to the cluster-level threshold (p < 0.00625).

*FC* functional connectivity, *DCM* degenerative cervical myelopathy, *CMCT* central motor conduction time, *SEP* somatosensory evoked potential, *R* right, *L* left, *BA* Brodmann area.

### Correlation between FC strength and SEP amplitude in patients with DCM

**Figure 2:**
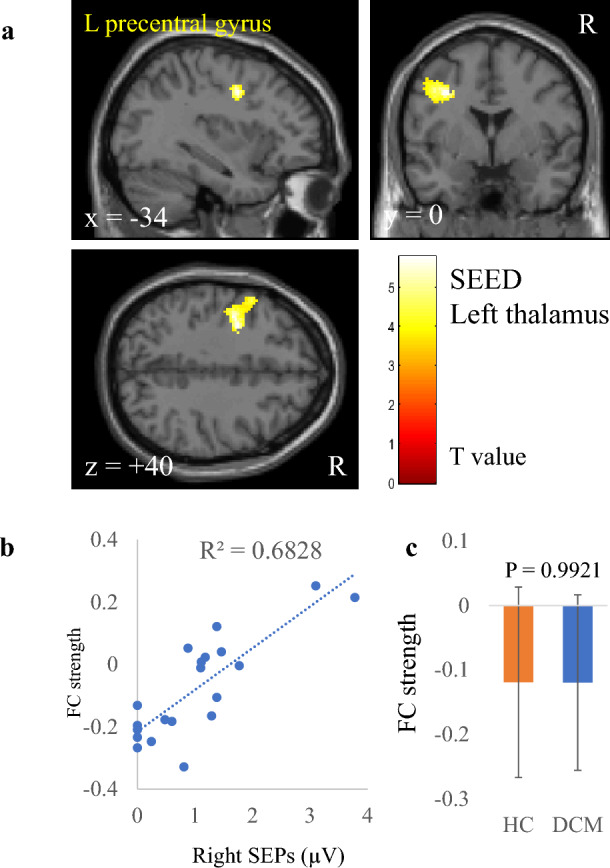
Correlation between right somatosensory evoked potentials (SEP) amplitude and functional connectivity (FC) strength of the left thalamus. (**a**) A cluster showing a significant positive correlation between right SEP and FC strength of the left thalamus, (**b**) scatter plot of right SEP versus FC strength, and (**c**) comparison of FC strengths between patients with degenerative cervical myelopathy (DCM) and healthy controls (HC). The dashed line in panel (**b**) is the regression line. Error bars in panel (**c**) represent standard deviations.

**Figure 3:**
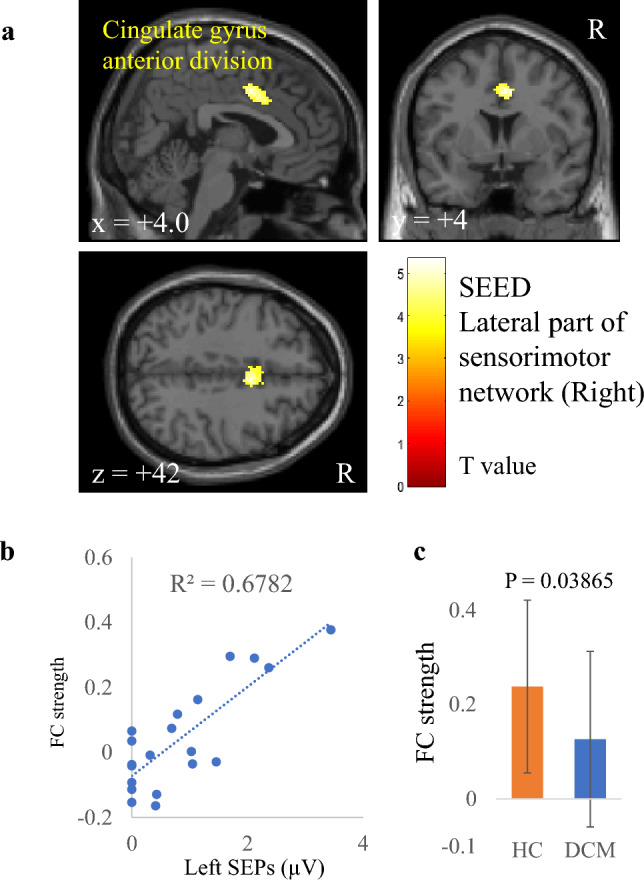
Correlation between left somatosensory evoked potentials (SEP) amplitude and functional connectivity (FC) strength of the right lateral part of the sensorimotor network. (**a**) A cluster showing a significant positive correlation between left SEP and FC strength of the right lateral part of the sensorimotor network, (**b**) scatter plot of left SEP versus FC strength, and (**c**) comparison of FC strengths between patients with degenerative cervical myelopathy (DCM) and healthy controls (HC). The dashed line in panel (**b**) is the regression line. Error bars in panel **c** represent standard deviations.

**Figure 4:**
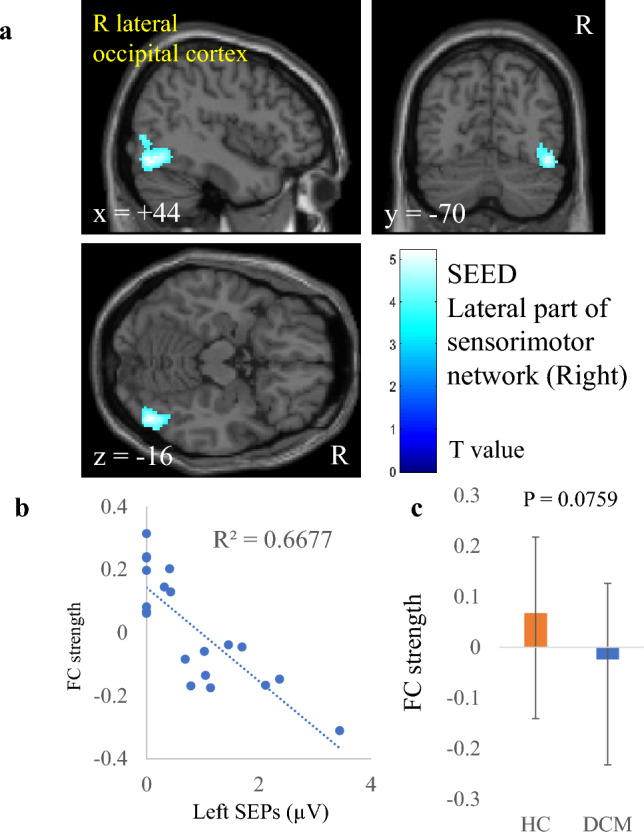
Correlation between left somatosensory evoked potentials (SEP) amplitude and functional connectivity (FC) strength of the right lateral part of the sensorimotor network. (**a**) A cluster showing a significant negative correlation between left SEP and FC strength of the right lateral part of the sensorimotor network, (**b**) scatter plot of left SEP versus FC strength, and **c**) comparison of FC strengths between patients with degenerative cervical myelopathy (DCM) and healthy controls (HC). The dashed line in panel (**b**) is the regression line. Error bars in panel (**c**) represent standard deviations.

As with CMCT, FC strength between the left thalamus and the left precentral gyrus also correlated significantly and positively with right SEP amplitude (r = 0.826, Fig. 2, Table 2). However, the two groups had no significant difference in this FC strength (p = 0.9921). FC strength between the sensorimotor lateral and cingulate gyrus anterior division also correlated with left SEP amplitude (r = 0.824, Fig. 3, Table 2). Moreover, the difference in strength of this FC was significant between the patients with DCM and the HC (p = 0.03865). In contrast, although FC strength between the sensorimotor lateral and the lateral occipital cortex correlated with left SEP amplitude (r = 0.817, Fig. 4, Table 2), the difference in this FC strength was not significant between the two groups (p = 0.0759).

## Discussion

In the present study, we attempted to apply rs-fMRI to explore predictive measures for evaluating the severity of DCM. Previous rs-fMRI studies have reported several correlations between brain activity indices, such as FC, ALFF, and regional homogeneity, and clinical scores/scales or neurological symptoms^31,35,37^. To our best knowledge, however, this is the first rs-fMRI study to show a correlation between the electrophysiological indices and FC strength in patients with DCM.

This study showed a significant increase in fALFF in the occipital pole and lateral occipital cortex superior division in the patients with DCM. A previous study also noted that patients with DCM showed an increase in fALFF in the occipital cortex^33^. Interactions between visual and proprioceptive information have an important role in motor programming^56,57^. Proprioception is impaired in patients with DCM^58^, and severe DCM leads to severe movement disorders. Therefore, this increase in fALFF in the occipital cortex suggests that the visual system compensates for the lack of proprioceptive information. We also found a significant decrease in fALFF in the cerebellar hemisphere, superior parietal lobule, frontal pole, right central opercular cortex, thalamus, and the inferior temporal gyrus posterior division. These brain areas are thought to taken part in sensory information processing and/or cognitive function. A previous study revealed that patients with DCM have sensory and cognitive deficits^42^, therefore we think that the decrease in fALFF in these areas reflects such functional deficits in patients with DCM.

CMCT is an objective measure representing the condition of the central motor pathway. In the present study, FC strength between the primary motor cortex and the precuneus correlated positively with CMCT (Fig. 1). The role of the primary motor cortex in the execution of movements is crucial^59^. In addition, the precuneus, a part of the default mode network, is related to performing a variety of highly integrated cognitive tasks involving visuo-spatial imagery, retrieval of episodic memory, and self-processing operations^60,61^. As patients with severe DCM have severe movement disorders, it can be assumed that patients with severe DCM need to functionally compensate for impaired motor functions to maintain their motor ability^33,62^. The role of the precuneus in integrating sensorimotor and other endo/exogenous information is also crucial^63^. That is, the large-scale brain network may interact with the primary motor cortex via the precuneus, and this interaction may be involved in the cognitive compensation for motor dysfunction. Klöppel et al. reported that the interaction between executive and cognitive motor areas, i.e., the supplementary motor area and superior parietal lobule, respectively, was involved in compensation for motor deficits of patients with pre-symptomatic Huntington’s disease^64^. In addition, another previous study revealed that sensorimotor network-default mode network interactions were involved in the compensatory function of motor performances in the stroke rehabilitation process^65^. Considering these findings, brain regions involved in cognitive functions may assist processing in motor areas and compensation for motor deficits. Therefore, the increased FC strength between the primary motor cortex and the precuneus in patients with DCM may reflect cognitive compensation of motor dysfunction in these patients.

SEP amplitude is an objective measure for the assessment of the condition of the sensory tracts or sensory loss. FC strength between the thalamus and the precentral gyrus anterior division correlated with SEP amplitude in this study (Fig. 2). A previous study showed that a postoperative DCM group manifested decreased FC between the right thalamus and the bilateral primary motor cortex^66^. The primary motor cortex is crucially involved in executing voluntary movements. In addition, the thalamus works as relay hub that directs information between different subcortical areas and the cerebral cortex, especially in the sensory area. Both are parts of the sensorimotor network, which processes bodily sensations and sends signals to the motor cortex to execute appropriate motor responses. We think that the positive correlation between FC strength and SEP amplitude reflects functional compensation for the sensory and motor disability in patients with DCM^67^.

A correlation of FC strength between the sensorimotor lateral and the cingulate gyrus anterior division with right SEP amplitude was also found. Moreover, this was the only FC to show a significant group difference (Fig. 3). The anterior cingulate cortex is the central part of the salience network, which is thought to be associated with allocating attentional resources to a salient stimulus such as pain^68^. It is also well known that acute pain activates the salience network and the sensorimotor system^69^. In patients with DCM, the more severe the spinal cord injury, the more impaired is signal transmission from the body to the brain. As a result, severe sensory deficits may decrease FC strength between the sensorimotor and anterior cingulate cortex, thus causing dysfunction of sensory information integration in the sensorimotor network and the salience network in these patients^70^. Moreover, the significant difference in this FC strength between the patients with DCM and the HC further supports the notion that alteration of this FC is related to sensory deficits in these patients.

We also showed that FC strength between the sensorimotor lateral and lateral occipital cortex correlated with SEP amplitude (Fig. 4). Sensory deficits occur in not only exteroception but also in deep sensation^58,71^. Impairment of deep sensation probably causes visual dependence on motor control. Therefore, our finding reflects the visual information-dependent compensatory mechanisms for sensorimotor deficits of the upper limbs in patients with DCM^72,73^.

Although we observed significant correlations between several FC strengths and CMCT or SEP amplitude in DCM patients, we did not find significant differences between the DCM and HC among them except for one (FC between sensorimotor lateral and cingulate gyrus anterior division). The interpretation of the results is difficult because the relationship between the electrophysiological indices and FC strengths has never been investigated in humans or animals.

We speculate there are two reasons for the lack of difference in FC strengths between the DCM and HC. One reason is the physical status during rs-fMRI scans, and the other is that tract damage indirectly affects FC strengths. DCM patients felt little neurological deficit during rs-fMRI scans because they did not perform active movements of the upper and lower limbs. Therefore, spontaneous brain activities, particularly FC, of patients with DCM may have not differed from those of HCs. The other reason is that damage of the pyramidal tract (reflected by CMCT) and the posterior funiculus (reflected by SEP) indirectly affect FC strengths in patients with DCM we revealed the significant correlations in this study. The changes in FC strengths in patients with DCM are thought to reflect the compensatory mechanisms for maintenance of motor and sensory functions depending on visual information and cognitive functions. Therefore, changes in FC strengths in patients with DCM may be too small to show significant differences to HCs, even though they show significant correlations to physiological measures. Further studies for not only humans but also animals are necessary in order to test these speculations.

### Limitations

Several patients with DCM had been administered analgesia. Previous studies revealed that analgesic agents acting on the central nervous system affect resting brain activity^74,75^. Therefore, analgesics may have influenced the present study results. Information on the analgesics administered in each patient is shown in Table 3.

**Table 3:** Characteristics of the patients.

Subject no	Sex (male/female)	Age (years)	CMCT data (+/−)	SEP data (+/−)	Medication	Disease duration (months)	JOA score						
							Motor dysfunction of upper extremity	Motor dysfunction of lower extremity	Sensory deficit of upper extremity	Sensory deficit of lower extremity	Sensory deficit of trunk	Bladder function deficit	Total
1	F	49	+	+	NSAIDs	11	3	4	1	2	1	3	14
2	F	52	+	−	NSAIDs	3	4	4	0.5	2	2	3	15.5
3	F	55	+	−	None	11	1	0	0.5	2	2	0	5.5
4	F	60	+	−	None	10	2	4	1	2	2	3	14
5	F	65	+	−	None	132	2	2	1	1	1	3	10
6	F	68	+	+	None	12	1	1	1	2	1	3	9
7	F	74	+	+	PG, TR, AC	1	1	0.5	0.5	0.5	0.5	3	6
8	F	79	+	+	None	36	1	1	1	1	1	3	8
9	F	80	−	−	None	6	2	2	1.5	2	1.5	2	11
10	F	82	+	−	NSAIDs	5	2	1.5	1.5	2	2	3	12
11	F	85	+	+	NSAIDs, TR, AC	7	2	1.5	1	1.5	1.5	3	10.5
12	M	33	+	+	None	26	3	1	1	2	2	3	12
13	M	45	+	+	NSAIDs, PG	3	2	1	1	1	0	3	8
14	M	49	+	+	NSAIDs	4	2	1	1	1	1	3	9
15	M	52	+	+	TR, AC	6	3	3	1	2	1	1	11
16	M	54	+	+	NSAIDs, TR, AC	48	4	4	1	2	1	3	15
17	M	54	+	+	None	13	1	4	1	2	1	3	12
18	M	56	+	+	None	60	3	4	1.5	2	1.5	3	15
19	M	62	+	+	None	3	2	2	1	2	1.5	1	9.5
20	M	62	+	−	PG, TR, AC	2	3	2.5	1	1	1	3	11.5
21	M	64	+	+	None	180	3	0	1.5	2	1.5	3	11
22	M	65	+	−	None	10	2	2.5	1	2	2	3	12.5
23	M	65	+	+	None	24	1	1.5	1	2	0.5	2	8
24	M	67	+	−	None	7	4	1	2	1	0	3	11
25	M	68	+	+	None	36	2	1	1	1	1	3	9
26	M	69	+	−	NSAIDs, PG	17	2	2	1	2	1.5	3	11.5
27	M	69	+	+	None	98	1	3	1	2	2	3	12
28	M	71	+	+	None	10	3	1	1	2	2	3	12
29	M	74	+	+	None	12	2	0.5	0.5	2	2	3	10
30	M	77	+	−	NSAIDs	60	2	2.5	1	2	1	3	11.5
31	M	79	+	−	None	12	3	4	1.5	2	2	2	14.5
32	M	80	+	−	NSAIDs, PG	19	1	2	2	2	2	3	12
33	M	83	+	+	NSAIDs	24	2	1.5	0.5	2	1.5	3	10.5
34	M	83	+	−	NSAIDs, PG	10	2	2	0.5	2	1	3	10.5

*CMCT* central motor conduction time, *SEP* somatosensory evoked potential, + available, − unavailable, *NSAIDs* nonsteroidal anti-inflammatory agents, *PG* pregabalin, *TR* tramadol, *AC* N-acetyl-p-aminophenol, *JOA score* Japanease Orthopaedic Association score.

Although SEP amplitude is a continuous variable, the SEP amplitudes were zero in 14 cases due to severe sensory dysfunction. Therefore, the “floor effect” may have affected the results of the correlation analysis. Intraoperative SEP examination was conducted under anesthesia. From the viewpoint of how alterations of SEPs in patients with DCM affect their brain activities, this is also the limitation in this study because the conditions of patients differ between the rs-fMRI scans and SEP measurements. However, in clinical, SEPs measured under anesthesia are widely used. Moreover, as mentioned above, SEP examination places a burden on patients. Considering them, rs-MRI without such a burden is the reasonable method to develop alternative SEP measures which detect sensory tract damage, and we believe that it is reasonable to compare rs-fMRI measures in awake rest and SEPs under anesthesia.

In this study we only revealed correlations between FC strength and preoperative electrophysiological measures. As is widely known, correlation does not imply causation. Moreover, we did not validate the accuracy of FC as a predictive measure of the severity of DCM. Therefore, it will be necessary to evaluate the capability of FC to assess DCM with larger samples in future studies.

## Conclusion

We showed significant correlations between FC strengths among certain brain regions and the preoperative and intraoperative neurophysiological indices of CMCT and SEP amplitude in patients with DCM. Our findings indicate the feasibility of rs-fMRI and FC analysis to provide novel predictive measures for assessment of the neurological severity of DCM.

## Materials and methods

### Participants

We recruited 34 patients with DCM (11 females, mean age: 65.6 years, range: 33–85 years) at our institution from May 2020 to June 2021. Inclusion criteria were that they (1) agreed to participate in this study; (2) had a medical indication for cervical decompression surgery; and (3) had symptoms caused by DCM^6,7^. Exclusion criteria were that they (1) refused to enroll; (2) had traumatic spinal cord compression; and (3) had a history of brain diseases such as cerebrovascular disease or tumors. We also recruited 21 age- and sex-matched HC, (7 females, mean age: 65.6 years, range: 33–83 years). The characteristics of the patients with DCM are shown in Table 3. We used the Japanese Orthopedic Association (JOA) score to assess DCM severity. The JOA score consists of six domains: motor function of upper extremity/lower extremity, sensory deficit of upper extremity/lower extremity/trunk, and bladder dysfunction^76^.

The Ethical Review Board of Yamaguchi University Hospital approved all protocols for this study (approval no.: H30-207-2), which was performed in accordance with approved guidelines and in compliance with the principles of the Declaration of Helsinki. All participants provided written informed consent before undergoing any study procedure.

### Electrophysiological assessment

We conducted an electrophysiological examination to assess objective neurological function and deficit in each patient with DCM. We measured preoperative CMCT in the upper and lower limbs and intraoperative posterior tibial nerve SEP.

### Measurement of CMCT

We examined the CMCT in all of the patients with DCM. We placed self-adhesive surface recording electrodes on the target muscles according to the belly-tendon method. The motor evoked potentials (MEPs) were recorded on the left side during voluntary contraction from the abductor digiti minimi (ADM) and abductor hallucis (AH) muscles^72^. We delivered transcranial magnetic stimulation through a round coil of 14-cm outer diameter (Magstim Co. Ltd., Spring Gardens, Whitland, UK) positioned flat on the scalp and centered over the Cz in the International 10–20 system at an intensity set at 20% above the MEP threshold during voluntary contraction.

We first recorded the electromyogram (EMG) during maximum voluntary contraction. Then, the patients with DCM were told about the amplitude of their raw EMG signal, and they worked to maintain a level 10–20% of that recorded during the maximum voluntary contraction. With this procedure, we confirmed that patients could maintain 10–20% of the maximal force of contraction during the MEPs measurements. A supramaximal electric stimulation was delivered to the ulnar nerve at the wrist and the tibial nerve at the ankle during which compound muscle action potentials (CMAPs) and F waves were recorded. Reference ground plates were located on the forearm or lower limb. We examined 16 serial responses and measured the shortest latency of the F waves. CMCT was calculated as MEPs latency − (CMAPs latency + F wave latency − 1)/2 (ms). All CMCT measurements were made with a Nicolet Viking 4 instrument (Natus Medical Incorporated, San Carlos, CA, USA). CMCT-ADM and CMCT-AH were measured in all patients^39,78^.

### Measurement of SEP

We examined intraoperative posterior tibial nerve (PTN) SEP in 20 patients with DCM. For intraoperative neuromonitoring, patients were placed under total intravenous anesthesia maintained with propofol 2.5–3.5 mg/mL delivered via a target-controlled infusion technique and remifentanil 0.1–0.3 mg/kg/min. Rocuronium 0.6–0.9 mg/kg (muscle relaxant) was administered only at the induction of anesthesia. The bispectral index score (BIS) derived from a frontal electroencephalogram (Aspect Medical Systems, Newton, MA, USA) was used to monitor anesthetic depth. An anesthetic depth associated with 40 < BIS < 60 was maintained by adjusting the rate of propofol infusion.

Stimulation needs to be delivered to the posterior tibial nerve at the ankle. Surface electrodes were placed on the skin over the nerve where it passes posterior to the medial malleolus. The cathode was applied midway between the medial border of the Achilles tendon and posterior border of the malleolus, whereas the anode electrode was applied away from the nerve 3 cm distal to the cathode. The electrical pulse delivered should have sufficient intensity to cause plantar flexion of the toes of 1–2 cm. The stimulator was set at intensity 30–40 mA, stimulus rate 4.7 Hz, and duration 0.5 ms according to the Guidelines of the International Federation of Clinical Physiology^79^. We used the same stimulation system with all patients. PTN-SEPs were recorded in a 60–80-ms time window following the stimulation. The electrode for scalp recording was placed at site Cz of the international 10–20 system, and the reference electrode was located at the Fz site on the forehead, and electrical impedances at these sites were < 5000 Ω. SEP amplitude was defined as the peak-to-peak amplitude of N1 and P1 (I-PTN). The amplifier settings for I-PTN-SEP recording included 20–3000-Hz bandpass filtering and 60-Hz notch filtering, with 200 trials averaged^44^. All SEP tests were performed with a Neuromaster MEE-1200 (Nihon Kohden, Tokyo, Japan). Senior electrophysiologists with 10 years of experience in evoked potentials study analyzed the recordings for presence of the main peaks P1-N1.

### MRI data acquisition

MRI scanning was by a 3.0 Tesla MRI scanner (Siemens Magnetom Prisma, Siemens Healthineers, Erlangen, Germany). During scanning, participants were instructed to simply rest with their eyes open, stare at one point, not to think of anything, and try not to fall asleep. We also confirmed whether the participants had not fallen asleep during the resting scan by direct questioning. To acquire the functional images, a 10-min scan was performed using a gradient-echo echo-planar pulse sequence with the following parameters: repetition time = 2500 ms, echo time = 30 ms, slice thickness = 3.2 mm, field of view = 212 × 212 mm, number of slices = 40, acquisition order = ascending, matrix size = 64 × 64, flip angle = 80°, and number of volumes = 245. Three-dimensional T1-weighted anatomical images were additionally acquired using magnetization-prepared acquisition with gradient echo sequence (MPRAGE; repetition time = 1800 ms, echo time = 2.88 ms, slice thickness = 1 mm, field of view = 256 × 256 mm, number of slices = 176, matrix size = 256 × 256, and flip angle = 10°).

### Data quality assessment

To check data quality, we confirmed no differences in the number of excluded fMRI volumes and size of body movements in each direction (x, y, z, pitch, roll, yaw) between the two groups by using two-sample *t*-tests. The thresholds were set at p < 0.05 for these tests.

### rs-MRI data analysis

#### Preprocessing

Data pre-processing was done using the CONN-fMRI toolbox (version 20b)^80^ for Statistical Parametric Mapping (SPM) 12 (revision number 7771, http://www.fil.ion.ucl.ac.uk/spm/, Wellcome Department of Cognitive Neurology, University College London, London, UK)^76^ implemented in MATLAB 2022a (Math Works, Natick, MA, USA).

The first five volumes of each participant’s fMRI data were discarded, and then the following steps were performed: slice timing correction, realignment, normalization to the Montreal Neurological Institute stereotactic template with resolution of 2 × 2 × 2-mm cubic voxels and smoothing with 8-mm full-width at half-maximum Gaussian kernel.

In addition to image processing, to remove non-neural artifacts, components associated with fMRI signals in white matter/cerebrospinal fluid were extracted with the component-based noise correction procedure^82^, after which six motion parameters were regressed from the data. Subsequently, scrubbing of high-motion timepoints was also performed, and finally, a 0.008–0.09-Hz bandpass filter was applied^83,84^.

#### fALFF analysis

For each participant, we calculated the fALFF value^54^ in each voxel of preprocessed rs-fMRI data using the CONN-fMRI toolbox and SPM12. ALFF value is a measure of BOLD signal power within the low frequency band (typically 0.01–0.1 Hz), and is defined as the root mean square of the BOLD signal at each voxel after temporal filtering^55^. fALFF value is a relative measure of BOLD signal power within the low frequency band compared to the entire frequency spectrum, and is defined as the ratio of root mean square of the BOLD signal at each voxel after vs. before temporal filtering^54^. Although these indices have been developed to characterize regional spontaneous brain activity, fALFF has been proven to be more gray matter-specific and sensitive to BOLD signal^14^.

#### FC analysis

We conducted seed-to-voxel correlation analyses with the CONN-fMRI toolbox and SPM12 to evaluate FC in the patients with DCM and HC. This analysis computed functional connectivity between a seed and remaining voxel as the Fisher-transformed bivariate correlation coefficients between a seed BOLD signal and each voxel BOLD signal.

We used the brain region related to the spinothalamic and pyramidal tracts, i.e., the thalamus, primary somatosensory cortex, and primary motor cortex, as seeds^85–89^. The seed templates came from the CONN toolbox.

### Statistics

We performed a two-sample t-test to compare fALFF values between CM and HC and a multiple regression analysis to investigate the relationship between fALFF values and electrophysiological measures. In multiple regression analysis, covariates of interest were right CMCT, left CMCT, right SEP amplitude, and left SEP amplitude. Moreover, age, disease duration, and JOA score was put into the model as covariates of uninterest. For these statistical tests, the thresholds were set at p < 0.001 (uncorrected, peak-level) and p < 0.05 (false discovery rate [FDR]-corrected, cluster-level). Furthermore, multiple comparison correction (Bonferroni correction) was applied to the cluster level threshold in the correlation analyses (p < 0.0125).

To investigate the relationship between FC strengths and electrophysiological measures, we performed multiple regression analysis. As in the multiple regression analysis for fALFF values, we put right CMCT, left CMCT, right SEP amplitude, or left SEP amplitude into the regression model as covariates of interest, and put age, disease duration, and JOA score into the model as covariates of uninterest. In particular, for FC of the left or right primary motor cortex, contralateral CMCT was a covariate of interest. For FC of the left or right primary somatosensory cortex or thalamus, contralateral SEP amplitude was a covariate of interest.

The thresholds were set at p < 0.001 (uncorrected, peak-level) and p < 0.05 (FDR-corrected, cluster-level). Multiple comparison correction (Bonferroni correction) was further applied to cluster-level threshold values (p < 0.00625). Moreover, for FC showing a significant correlation in the correlation analysis, we compared its strength between patients with DCM and HC using a two-sample *t*-test. The thresholds were set at p < 0.05 for these tests.

We created bar graphs and scatter plots with Microsoft Excel version 16.73 and used its functions to add error bars to the bar graphs and regression lines to the scatter plots and to calculate the coefficients of determination (R^2^). Analysis of all data was performed with StatFlex Ver. 7 for Windows (Artec, Osaka, Japan; https://www.statflex.net/).

### Supplementary Information

Supplementary Table 1.

## Data Availability

The datasets obtained and/or analyzed in the present study are available from the corresponding author on reasonable request.

## References

[1] Nouri, A., Tetreault, L., Singh, A., Karadimas, S. K. & Fehlings, M. G. Degenerative cervical myelopathy: Epidemiology, genetics, and pathogenesis. *Spine (Phila. Pa. 1976)* **40**, 675–693 (2015). 10.1097/BRS.0000000000000913 25839387

[2] Nouri A (2020). Degenerative cervical myelopathy: A brief review of past perspectives, present developments, and future directions. J. Clin. Med..

[3] Fehlings MG (2017). A clinical practice guideline for the management of patients with degenerative cervical myelopathy: recommendations for patients with mild, moderate, and severe disease and nonmyelopathic patients with evidence of cord compression. Global Spine J..

[4] Karadimas, S. K., Erwin, W. M., Ely, C. G., Dettori, J. R. & Fehlings, M. G. Pathophysiology and natural history of cervical spondylotic myelopathy. *Spine (Phila. Pa. 1976)* **38**, 21–36 (2013). 10.1097/BRS.0b013e3182a7f2c3 23963004

[5] Tetreault L (2015). Degenerative cervical myelopathy: A spectrum of related disorders affecting the aging spine. Neurosurgery.

[6] Kalsi-Ryan S, Karadimas SK, Fehlings MG (2013). Cervical spondylotic myelopathy: The clinical phenomenon and the current pathobiology of an increasingly prevalent and devastating disorder. Neuroscientist.

[7] Harrop, J. S. *et al*. Cervical myelopathy: A clinical and radiographic evaluation and correlation to cervical spondylotic myelopathy. *Spine (Phila. Pa. 1976)* **35**, 620–624 (2010). 10.1097/BRS.0b013e3181b723af 20150835

[8] Machino, M. *et al*. The prevalence of pre- and postoperative symptoms in patients with cervical spondylotic myelopathy treated by cervical laminoplasty. *Spine (Phila. Pa. 1976)* **37**, 1383–1388 (2012). 10.1097/BRS.0b013e3182684c68 22789979

[9] Acharya, S., Srivastava, A., Virmani, S. & Tandon, R. Resolution of physical signs and recovery in severe cervical spondylotic myelopathy after cervical laminoplasty. *Spine (Phila. Pa. 1976)* **35**, 1083–1087 (2010). 10.1097/BRS.0b013e3181df1a8e 20838272

[10] Seichi, A. *et al*. Neurologic level diagnosis of cervical stenotic myelopathy. *Spine (Phila. Pa. 1976)* **31**, 1338–1343 (2006). 10.1097/01.brs.0000219475.21126.6b 16721296

[11] Matsumoto M (2005). Usefulness of neurological examination for diagnosis of the affected level in patients with cervical compressive myelopathy: Prospective comparative study with radiological evaluation. J. Neurosurg. Spine.

[12] Nardone R (2016). The contribution of neurophysiology in the diagnosis and management of cervical spondylotic myelopathy: A review. Spinal Cord.

[13] Fujimoto, K. *et al*. Use of central motor conduction time and spinal cord evoked potentials in the electrophysiological assessment of compressive cervical myelopathy. *Spine (Phila. Pa. 1976)* **42**, 895–902 (2017). 10.1097/BRS.0000000000001939 27792117

[14] Nagata K (2014). The prevalence of cervical myelopathy among subjects with narrow cervical spinal canal in a population-based magnetic resonance imaging study: The Wakayama Spine Study. Spine J..

[15] Nouri A, Martin AR, Mikulis D, Fehlings MG (2016). Magnetic resonance imaging assessment of degenerative cervical myelopathy: A review of structural changes and measurement techniques. Neurosurg. Focus.

[16] Hori M (2014). Cervical spondylosis: Evaluation of microstructural changes in spinal cord white matter and gray matter by diffusional kurtosis imaging. Magn. Reson. Imaging.

[17] Matsumoto, M. *et al*. Increased signal intensity of the spinal cord on magnetic resonance images in cervical compressive myelopathy: Does it predict the outcome of conservative treatment? *Spine (Phila. Pa. 1976)* **25**, 677–682 (2002). 10.1097/00007632-200003150-00005 10752098

[18] Matsuda Y (1991). Increased MR signal intensity due to cervical myelopathy: analysis of 29 surgical cases. J. Neurosurg..

[19] Wang, K. *et al.* Evaluation of DTI parameter ratios and diffusion tensor tractography grading in the diagnosis and prognosis prediction of cervical spondylotic myelopathy. *Spine (Phila. Pa. 1976)* **42**, E202–E210 (2017). 10.1097/BRS.0000000000001784 28207659

[20] Chagawa K (2015). Normal values of diffusion tensor magnetic resonance imaging parameters in the cervical spinal cord. Asian Spine J..

[21] Suetomi Y (2016). Application of diffusion tensor imaging for the diagnosis of segmental level of dysfunction in cervical spondylotic myelopathy. Spinal Cord.

[22] Jin R (2019). Prognosis of cervical myelopathy based on diffusion tensor imaging with artificial intelligence methods. NMR Biomed..

[23] Schilling KG (2019). Limits to anatomical accuracy of diffusion tractography using modern approaches. Neuroimage..

[24] Baliki MN, Mansour AR, Baria AT, Apkarian AV (2014). Functional reorganization of the default mode network across chronic pain conditions. PLoS One.

[25] Wang C, Laiwalla A, Salamon N, Ellingson BM, Holly LT (2020). Compensatory brainstem functional and structural connectivity in patients with degenerative cervical myelopathy by probabilistic tractography and functional MRI. Brain Res..

[26] Kowalczyk I, Duggal N, Bartha R (2012). Proton magnetic resonance spectroscopy of the motor cortex in cervical myelopathy. Brain.

[27] Duggal N (2010). Brain reorganization in patients with spinal cord compression evaluated using fMRI. Neurology.

[28] Zhou FQ (2015). Intrinsic functional plasticity of the sensory-motor network in patients with cervical spondylotic myelopathy. Sci. Rep..

[29] Woodworth DC, Holly LT, Salamon N, Ellingson BM (2018). Resting-state functional magnetic resonance imaging connectivity of the brain is associated with altered sensorimotor function in patients with cervical spondylosis. World Neurosurg..

[30] Oni-Orisan A (2016). Alterations in cortical sensorimotor connectivity following complete cervical spinal cord injury: A prospective resting-state fMRI study. PLoS One.

[31] Kaushal M (2017). Evaluation of whole-brain resting-state functional connectivity in spinal cord injury: A large-scale network analysis using network-based statistic. J. Neurotrauma.

[32] Eto F (2022). Postoperative changes in resting state functional connectivity and clinical scores in patients with cervical myelopathy. World Neurosurg..

[33] Takenaka S (2020). Resting-state amplitude of low-frequency fluctuation is a potentially useful prognostic functional biomarker in cervical myelopathy. Clin. Orthop. Relat. Res..

[34] Wang C, Ellingson BM, Oughourlian TC, Salamon N, Holly LT (2022). Evolution of brain functional plasticity associated with increasing symptom severity in degenerative cervical myelopathy. EBioMedicine.

[35] Tan Y (2015). Alteration of regional homogeneity within the sensorimotor network after spinal cord decompression in cervical spondylotic myelopathy: A resting-state fMRI study. Biomed. Res. Int..

[36] Zhao G, Zhang C, Zhan Y, He L (2022). The correlation between functional connectivity of the primary somatosensory cortex and cervical spinal cord microstructural injury in patients with cervical spondylotic myelopathy. Dis. Markers.

[37] Takenaka S (2019). Towards prognostic functional brain biomarkers for cervical myelopathy: A resting-state fMRI study. Sci. Rep..

[38] Zhou F (2014). Increased low-frequency oscillation amplitude of sensorimotor cortex associated with the severity of structural impairment in cervical myelopathy. PloS One.

[39] Li X (2020). Inconsistency between cortical reorganization and functional connectivity alteration in the sensorimotor cortex following incomplete cervical spinal cord injury. Brain Imaging Behav..

[40] Su Q (2021). Identification and therapeutic outcome prediction of cervical spondylotic myelopathy based on the functional connectivity from resting-state functional MRI data: A preliminary machine learning study. Front. Neurol..

[41] Chen Z (2018). Visual cortex neural activity alteration in cervical spondylotic myelopathy patients: A resting-state fMRI study. Neuroradiology.

[42] Zhao R (2020). Neural correlates of cognitive dysfunctions in cervical spondylotic myelopathy patients: A resting-state fMRI study. Front. Neurol..

[43] Imajo Y (2021). Assessment of spinal cord relative vulnerability in C4–C5 compressive cervical myelopathy using multi-modal spinal cord evoked potentials and neurological findings. J. Spinal Cord Med..

[44] Imajo Y (2022). The reference intervals of intraoperative posterior tibial nerve somatosensory evoked potentials. J. Orthop. Sci..

[45] Funaba M (2014). Preoperative diagnosis of the responsible level in CCM using CMAPs: Comparison with SCEPs. Spinal Cord.

[46] Funaba, M. *et al*. Transcranial magnetic stimulation in the diagnosis of cervical compressive myelopathy: comparison with spinal cord evoked potentials. *Spine (Phila. Pa. 1976)* **40**, 161–167 (2015). 10.1097/BRS.0000000000000698 25384053

[47] Kanchiku T (2016). Correlation between spinal cord function assessed by intraoperative SCEPs and morphology of the compressed spinal cord on MRI. Clin. Spine Surg..

[48] Imajo Y (2011). Relative vulnerability of various spinal tracts in C3–4 cervical spondylotic myelopathy: Multi-modal spinal cord evoked potentials. Spinal Cord.

[49] Imajo, Y. *et al*. Prediction of surgical outcome for proximal-type cervical spondylotic amyotrophy novel mode of assessment using compound action potentials of deltoid and biceps brachii and central motor conduction time. *Spine (Phila. Pa. 1976)* **37**, 1444–1449 (2012). 10.1097/BRS.0b013e31826e2ead 22895483

[50] Rikita T (2017). The relationship between central motor conduction time and spinal cord compression in patients with cervical spondylotic myelopathy. Spinal Cord.

[51] Feng, X., Hu, Y. & Ma, X. Progression prediction of mild cervical spondylotic myelopathy by somatosensory-evoked potentials. *Spine (Phila. Pa. 1976)* **45**(10), E560–E567 (2020). 10.1097/BRS.0000000000003348 31770314

[52] Nakai S, Sonoo M, Shimizu T (2008). Somatosensory evoked potentials (SEPs) for the evaluation of cervical spondylotic myelopathy: Utility of the onset-latency parameters. Clin. Neurophysiol..

[53] Reddy RP (2021). What is the predictive value of intraoperative somatosensory evoked potential monitoring for postoperative neurological deficit in cervical spine surgery? A meta-analysis. Spine J..

[54] Zou QH (2008). An improved approach to detection of amplitude of low-frequency fluctuation (ALFF) for resting-state fMRI: Fractional ALFF. J. Neurosci. Methods.

[55] Yang H (2007). Amplitude of low frequency fluctuation within visual areas revealed by resting-state functional MRI. Neuroimage.

[56] Touzalin-Chretien P, Ehrler S, Dufour A (2010). Dominance of vision over proprioception on motor programming: evidence from ERP. Cereb. Cortex.

[57] Maunsell JHR (2015). Neuronal mechanisms of visual attention. Annu. Rev. Vis. Sci..

[58] Takayama, H. *et al*. Impaired joint proprioception in patients with cervical myelopathy. *Spine (Phila. Pa. 1976)* **30**(1), 83–86 (2005). 10.1097/00007632-200501010-00015 15626986

[59] Pearson K (2000). Motor systems. Curr. Opin. Neurobiol..

[60] Cavanna AE, Trimble MR (2006). The precuneus: a review of its functional anatomy and behavioural correlates. Brain.

[61] Raichle ME (2001). A default mode of brain function. Proc. Natl. Acad. Sci. U. S. A..

[62] Valera-Bermejo JM, De Marco M, Venneri A (2022). Altered interplay among large-scale brain functional networks modulates multi-domain anosognosia in early Alzheimer's disease. Front. Aging Neurosci..

[63] Margulies DS (2009). Precuneus shares intrinsic functional architecture in humans and monkeys. Proc. Natl. Acad. Sci. U. S. A..

[64] Klöppel S (2009). Functional compensation of motor function in pre-symptomatic Huntington's disease. Brain.

[65] Wu CW (2020). Synchrony between default-mode and sensorimotor networks facilitates motor function in stroke rehabilitation: A pilot fMRI study. Front. Neurosci..

[66] Peng X, Tan Y, He L, Ou Y (2020). Alterations of functional connectivity between thalamus and cortex before and after decompression in cervical spondylotic myelopathy patients: A resting-state functional MRI study. Neuroreport.

[67] Lv Q (2022). Somatosensory deficits after stroke: Insights from MRI studies. Front. Neurol..

[68] Uddin LQ (2015). Salience processing and insular cortical function and dysfunction. Nat. Rev. Neurosci..

[69] Apkarian AV, Bushnell MC, Treede RD, Zubieta JK (2005). Human brain mechanisms of pain perception and regulation in health and disease. Eur. J. Pain.

[70] Hegarty AK, Yani MS, Albishi A, Michener LA, Kutch JJ (2020). Salience network functional connectivity is spatially heterogeneous across sensorimotor cortex in healthy humans. Neuroimage.

[71] Boerger TF, McGinn L, Wang MC, Schmit BD, Hyngstrom AS (2022). Degenerative cervical myelopathy delays responses to lateral balance perturbations regardless of predictability. J. Neurophysiol..

[72] Bernard-Espina J, Beraneck M, Maier MA, Tagliabue M (2021). Multisensory integration in stroke patients: A theoretical approach to reinterpret upper-limb proprioceptive deficits and visual compensation. Front. Neurosci..

[73] Kuang C, Zha Y (2019). Abnormal intrinsic functional activity in patients with cervical spondylotic myelopathy: A resting-state fMRI study. Neuropsychiatr. Dis. Treat..

[74] Lee MC (2013). Amygdala activity contributes to the dissociative effect of cannabis on pain perception. Pain.

[75] Baliki MN (2008). A preliminary fMRI study of analgesic treatment in chronic back pain and knee osteoarthritis. Mol. Pain.

[76] Kato S (2015). Comparison of the Japanese Orthopaedic Association (JOA) score and modified JOA (mJOA) score for the assessment of cervical myelopathy: A multicenter observational study. PloS One.

[77] Corbetta M, Shulman GL (2002). Control of goal-directed and stimulus-driven attention in the brain. Nat. Rev. Neurosci..

[78] Imajo Y (2017). Effects of differences in age and body height on normal values of central motor conduction time determined by F-waves. J. Spinal Cord Med..

[79] MacDonald DB (2019). Recommendations of the International Society of Intraoperative Neurophysiology for intraoperative somatosensory evoked potentials. Clin. Neurophysiol..

[80] Whitfield-Gabrieli S, Nieto-Castanon A (2012). Conn: a functional connectivity toolbox for correlated and anticorrelated brain networks. Brain Connect..

[81] Friston KJ (1994). Statistical parametric maps in functional imaging: A general linear approach. Hum. Brain Mapp..

[82] Behzadi Y, Restom K, Liau J, Liu TT (2007). A component-based noise correction method (CompCor) for BOLD and perfusion based fMRI. Neuroimage.

[83] Weissenbacher A (2009). Correlations and anticorrelations in resting-state functional connectivity MRI: A quantitative comparison of preprocessing strategies. Neuroimage.

[84] De Pisapia N, Bacci F, Parrott D, Melcher D (2016). Brain networks for visual creativity: A functional connectivity study of planning a visual artwork. Sci. Rep..

[85] Fields H (2004). State-dependent opioid control of pain. Nat. Rev. Neurosci..

[86] Kolesar TA, Bilevicius E, Kornelsen J (2017). Salience, central executive, and sensorimotor network functional connectivity alterations in failed back surgery syndrome. Scand. J. Pain.

[87] Evarts EV (1968). Relation of pyramidal tract activity to force exerted during voluntary movement. J. Neurophysiol..

[88] Cheney PD, Fetz EE (1980). Functional classes of primate corticomotoneuronal cells and their relation to active force. J. Neurophysiol..

[89] Fox MD, Zhang D, Snyder AZ, Raichle ME (2009). The global signal and observed anticorrelated resting state brain networks. J. Neurophysiol..

