# Author Correction: Resting‑state functional magnetic resonance imaging indices are related to electrophysiological dysfunction in degenerative cervical myelopathy

**DOI:** 10.1038/s41598-024-60360-8

**Published:** 2024-04-25

**Authors:** Hironobu Akimoto, Hidenori Suzuki, Shigeyuki Kan, Masahiro Funaba, Norihiro Nishida, Kazuhiro Fujimoto, Hiroaki Ikeda, Teppei Yonezawa, Kojiro Ikushima, Yoichiro Shimizu, Toshio Matsubara, Kenichiro Harada, Shin Nakagawa, Takashi Sakai

**Affiliations:** 1 Department of Orthopedic Surgery, Yamaguchi University Graduate School of Medicine, 1‑1‑1 Minami‑Kogushi, Ube, Yamaguchi 755‑8505 Japan; 2 Department of Psychiatry and Neurosciences, Graduate School of Biomedical and Health Science, Hiroshima University, Hiroshima, Hiroshima 734‑8553 Japan; 3 Department of Anesthesiology and Intensive Care Medicine, Osaka University Graduate School of Medicine, Suita, Osaka 565‑0871 Japan; 4 Department of Radiological Technology, Yamaguchi University Hospital, 1‑1‑1 Minami‑Kogushi, Ube, Yamaguchi 755‑8505 Japan; 5 Division of Neuropsychiatry, Department of Neuroscience, Yamaguchi University Graduate School of Medicine, Ube, Yamaguchi 755‑8505 Japan

Correction to: *Scientific Reports* 10.1038/s41598-024-53051-x, published online 29 January 2024

The original version of this Article contained an error in the spelling of the author Toshio Matsubara which was incorrectly given as Toshiro Matsubara.

The original Article has been corrected.

